# Self-reported oral health in the Dutch 100-plus Study of cognitively healthy centenarians: an observational cohort study

**DOI:** 10.1186/s12877-019-1358-x

**Published:** 2019-12-18

**Authors:** Nina Beker, Claar D. van der Maarel-Wierink, Cees de Baat, Henne Holstege

**Affiliations:** 1 Alzheimer Center Amsterdam, Department of Neurology, Amsterdam Neuroscience, Vrije Universiteit Amsterdam, Amsterdam UMC, PO Box 7057, Amsterdam, 1007 MB The Netherlands; 2 BENECOMO, Flemish-Netherlands Geriatric Oral Research Group, Ghent, Belgium and Amsterdam, The Netherlands; 3 Center for Special Care in Dentistry, Amsterdam, The Netherlands; 4 Department of Oral Function and Prosthetic Dentistry, Radboud University Medical Center, Nijmegen, The Netherlands; 5 Department of Clinical Genetics, Amsterdam Neuroscience, Vrije Universiteit Amsterdam, Amsterdam UMC, Amsterdam, The Netherlands

**Keywords:** Oral health, 100-plus study, Centenarians, Longevity, Oldest-old

## Abstract

**Background:**

Due to improved healthcare, more people reach extreme ages. Oral health in the oldest-old has thus far been poorly described. Here, we investigated self-reported oral health factors, use of professional oral health care, and associations with clinical measures in centenarians considered cognitively healthy.

**Methods:**

In this observational cohort study, we included 162 (74% female) centenarians from the Dutch 100-plus Study cohort who self-reported to be cognitively healthy, as confirmed by a proxy. Centenarians were questioned about their physical well-being including medication use and their cognitive functioning was evaluated using the Mini-Mental State Examination. Questions regarding oral health included preservation of teeth, oral pain or discomfort, chewing ability, xerostomia, and time since last visit to an oral health care provider. Associations between oral health and clinical measures were investigated with ordinal logistic or linear regression analyses, adjusted for gender, age, and education.

**Results:**

The majority of the centenarians indicated to have good oral health: 76% felt no oral pain/discomfort, 65% indicated to chew well; while only 18% had symptoms of xerostomia. Of all centenarians, 83% were edentulous and were wearing removable complete maxillary and mandibular dental prostheses, 1% was edentulous with no dental prosthesis, while 16% was dentate with or without removable partial dental prostheses (10 and 6% respectively). Dentate and edentulous centenarians experienced similar levels of oral pain and/or discomfort, chewing ability, xerostomia, and their cognitive functioning was similar. No relationship between cognitive functioning and chewing ability was found. Xerostomia was associated with medication use (*p* = .001), which mostly regarded medications for cardiovascular diseases, diuretics, anti-coagulants, and antacids. Only 18% of the centenarians visited an oral health care provider during the year prior to the interview, of whom 48% were dentate centenarians. Notably, 49% of the centenarians had not visited an oral health care provider for ≥10 years.

**Conclusions:**

Most centenarians were edentulous and did not report oral complaints. Less than one-fifth of the centenarians continued to seek regular professional oral health care. Since the proportion of dentates in the oldest-old will increase in the near future, a proactive attitude toward this group is necessary.

## Background

Worldwide, the number of centenarians is estimated to increase eighteen fold by 2050, up to 3.2 million [1]. In the Netherlands, the number of centenarians is expected to double in the next twenty years [2]. Oral health has been shown to be an important predictor of longevity [3]. An unhealthy dentition increases the risk of oral inflammation, which associates with poor health and decreased life expectancy [4]. After controlling for life-style behaviors and health condition, the risk of all-cause mortality correlates with the number of natural teeth present, suggesting that the 5-year survival rate increased by 4% per retained tooth beyond the age of 70 years [5]. Other studies indicate that masticatory activity is a prerequisite for maintaining adequate cognitive functions [6, 7].

Factors with a potential negative effect on oral health are: no regular dental consultations and hyposalivation, often a consequence of medication use [8, 9]. This indicates that these factors should be considered when evaluating oral health.

Due to increasing longevity resulting from developments in general medical and oral health care, the number of dentate older people increases [10]. In fact, between 2000 and 2009, the fraction of the population in the Netherlands above 65 years wearing removable complete maxillary and mandibular dental prostheses decreased from 59 to 41% [11]. However, little is known about the oral health of people who reach extreme ages in cognitive health and to what extent they reach out to oral health care providers.

Here, we aim to describe the oral health and use of professional oral health care self-reported by cognitively healthy Dutch centenarians. In addition, we investigate associations between factors of oral health and use of prescription medications, cognitive functioning and independence in activities of daily living (ADL).

## Methods

### Study design, setting and participants

We investigated self-reported oral health in a subset of the centenarians included in the 100-plus Study. The 100-plus Study is an observational cohort study of Dutch centenarians (age ≥ 100 years) who self-report to be cognitively healthy, which is confirmed by a family member or caregiver*.* Centenarians were recruited by different media and mouth-to-mouth advertisement, as described previously [12]. Centenarians who were included between January 2013 and January 2016, (*n* = 162), were presented the oral health questionnaire. Visits took place at the home residence of the centenarians by trained researches with a neuropsychological and/or medical background.

### Data collection

Participants were interviewed about their (medical) history, lifestyle behavior and, current physical well-being including medication use [12]. Prescription medications were classified according to eleven medication-categories: cardiac medication, diuretics, anti-coagulants, laxatives, antacids, analgesic, sedatives, antidiabetics, antidepressants, iron supplements, and other. For each centenarian, we calculated the total number of categories for which they used at least one medication. Education level was categorized according to the International Standard Classification of Education 1997 (ISCED) [13]. Independence in activities of daily living (ADL) was assessed using the 10-item Barthel Index (BI) [14], with scores ranging from 0 to 20; scores < 15 indicate dependency and scores ≥15 indicate independence [15]. To assess cognitive functioning, we administered the Mini-Mental State Examination (MMSE) [16]: with scores ranging from 0 to 30; higher scores indicate better cognitive functioning [17]. We used multiple imputation to impute scores for missing items on the MMSE test attributable to vision and hearing disability [12]. In case less than 20% of the items were missing, we imputed total scores on the BI using mean imputation methods.

During the interview, centenarians were questioned about components of oral health which included the preservation of teeth, oral pain or discomfort, chewing ability, and xerostomia. We were informed about use of professional oral health care by asking the centenarians for the time period since the last visit to an oral health care provider; see Table 1 for the questionnaire. Xerostomia was assessed using the modified xerostomia questionnaire [18]; scores ≥8 indicated xerostomia.

**Table 1:** Questionnaire including the various components of oral health

Questions	
Do you have:	
☐ natural teeth ☐ natural teeth in combination with a removable partial denture ☐ removable complete dentures ☐ no natural teeth, no removable dentures	
What is the time period since your last visit to an oral health care provider?	
☐ Less than one year ☐ 1–5 year(s) ☐ 5–10 years ☐ 10–20 years	
Do you experience pain or discomfort in your mouth?	
☐ Yes ☐ No ☐ Sometimes	
Are you able to chew adequately?	
☐ Yes ☐ Rather good ☐ Poor ☐ No	
Which option does apply to your situation?*	
☐ My mouth feels dry when eating ☐ My mouth feels dry ☐ I have difficulty eating dry foods ☐ I have difficulties swallowing certain foods ☐ My lips feel dry	

*Xerostomia questionnaire. Reply on this questionnaire can be: “never” = 1, “sometimes” = 2, “always” = 3. A total sumscore < 8 indicates no xerostomia, a total sumscore ≥8 indicates xerostomia

### Analyses

Statistical analyses were performed using MASS package in R (v 3.5.2) (The R Foundation, https://www.r-project.org/). We investigated associations between oral health and clinical measures with ordinal logistic regression analyses for ordinal outcome variables and linear regression analyses for continuous outcome variables, adjusted for gender, age, and education. Statistics were presented by odds ratios (OR) or betas and 95% confidence intervals. A *p*-value of 0.05 was considered statistically significant.

## Results

### Demographic, clinical and oral health characteristics

In total, 354 centenarians were approached by January 2016, of whom 46 were not eligible because of cognitive impairment, 54 had no interest in participating, 26 found it too burdensome, 16 were deceased and 50 centenarians were not included for other or unknown reasons.

The 162 centenarians we investigated were born between 1902 and 1915; 74% were women, and 52% were educated at a medium level or higher. 50% of the centenarians were community-dwelling, 69% were mobile with or without aids, or with assistance of another person. Most centenarians were ADL independent (BI score ≥ 15: 49% vs BI score < 15: 38%) and had high cognitive functioning (median [IQR] MMSE score: 26 [23-28]). More clinical descriptives of this cohort of centenarians are represented in Table 2.

**Table 2:** Clinical and demographic characteristics (total *n* = 162)

Age, median (IQR)	100.9 (100.2–102.1)
Gender, female N (%)	120 (74)
Education, N (%)	
Low	76 (47)
Medium	62 (38)
High	23 (14)
Missing value	1 (1)
Cognitive functioning, MMSE, median (IQR)^a^	26 (23–28)
Missing value, N (%)	8 (5)
Living situation, N (%)	
Community-dwelling, or independent in a residence with available services	82 (50)
Fulltime assistance at home residence	3 (2)
In a residential care center	67 (41)
In a nursing home	3 (2)
With family	6 (4)
Missing value	1 (1)
Independence in ADL, BI median (IQR)^b^	15 (11–19)
Independent (scores ≥15), N (%)	79 (49)
Dependent (scores < 15), N (%)	61 (38)
Missing value	22 (13)
Mobility, N (%)	
Able to walk independently, with or without a walking stick or walker	112 (69)
Able to walk with help of another person	11 (7)
Able to move independently in a wheelchair	11 (7)
Not able to move independently in a wheelchair	9 (5)
Missing value	19 (12)
Prescription medication categories, median (IQR)	3 (5–7)
Cardiac medication, N (%)	86 (53)
Diuretics, N (%)	76 (47)
Anti-coagulants, N (%)	73 (45)
Antacids, N (%)	66 (41)
Analgesic, N (%)	41 (25)
Sedatives, N (%)	26 (16)
Laxatives, N (%)_	29 (18)
Antidiabetics, N (%)	8 (5)
Antidepressants, N (%)	4 (3)
Iron supplements, N (%)	11 (7)
Other, N (%)	80 (49)
Missing value	10 (6)
Disease history, yes N (%)	
Cardiovascular disease	86 (53)
Musculoskeletal disease	69 (43)
Sensory problems	49 (30)
Cancer	30 (19)
Autoimmune disease	10 (6)
Other	63 (39)
Missing value	55 (34)
Preservation of teeth, N (%)	
Edentulous/no removable complete dental prostheses	2 (1)
Dentate without removable partial dental prosthesis	10 (6)
Dentate with removable partial dental prosthesis	16 (10)
Edentulous and removable complete dental prostheses	134 (83)
Oral pain or discomfort, N (%)	
No	123 (76)
Sometimes	13 (8)
Yes	7 (4)
Missing value	19 (12)
Chewing ability, N (%)	
Good	106 (65)
Rather good	23 (14)
Poor	10 (6)
Not able	3 (2)
Missing value	20 (13)
Xerostomia, median (IQR)^c^	6 (5–7)
Yes (scores ≥8), N (%)	30 (18)
No (scores < 8), N (%)	108 (67)
Missing value	24 (15)
Time period since last visit to an oral health care provider, N (%)	
Less than 1 year	29 (18)
1 to 5 years	29 (18)
5 to 10 years	14 (8)
More than 10 years	79 (49)
Missing value	11 (7)

Values are presented by number (N) and percentage (%) or median and IQR. ^a^Score range of the MMSE: 0 to 30. Higer scores indicate better performance. ^b^Score range of the Barthel Index: 0 to 20. Higher scores indicate more independency in ADL. ^c^Score range of the xerostomia questionnaire: 5 to 15. Higher scores indicate more xerostomia symptoms. BI=Barthel Index. MMSE = Mini-Mental State Examination. ADL = Activities of daily living. IQR = Interquartile range. SD = Standard Deviation

### Oral health characteristics and use of professional oral health care

The large majority of the centenarians (*n* = 134, 83%), were edentulous and were wearing removable maxillary and mandibular complete dental prostheses; 2 centenarians (1%) were edentulous and did not wear removable complete dental prostheses; 10 centenarians (6%) were fully dentate and did not wear removable partial dental prostheses; 16 (10%) were dentate and were wearing one or two removable partial dental prostheses. By self-report, 123 centenarians (76%) did not experience oral pain or discomfort; 106 (65%) could chew adequately; for 30 centenarians (18%) xerostomia was assessed (by a score ≥ 8 on the xerostomia questionnaire).

Twenty-nine centenarians (18%) had visited an oral health care provider during the year prior to the interview, while 79 centenarians (49%) had not visited an oral health care provider for at least 10 years prior to the interview (Table 2).

### Self-reported oral health in dentate and edentulous centenarians

Participants were stratified as follows: (1) dentate with or without removable partial dental prostheses, denoted dentate centenarians, *n* = 26 (16%); (2) edentulous and wearing removable complete maxillary and mandibular dental prostheses, denoted edentulous centenarians, *n* = 134 (83%). The two edentulous centenarians who did not wear removable complete maxillary and mandibular dental prostheses were excluded from analyses on differences between these groups. Dentate and edentulous centenarians experienced similar levels of oral pain and/or discomfort (*p* = .809), and their chewing ability and xerostomia scores were similar (*p* = .896 and *p* = .877 respectively, Table 3). Both groups had similar levels of cognition (*p* = .753), and we observed no relationship between chewing ability and cognitive functioning (*p* = .623) (Table 3).

**Table 3:** Oral health factors associated with clinical measures in self-reported cognitively healthy centenarians (n = 162)

		Cognitive functioning		Oral pain or discomfort		Chewing ability		Use of professional oral health care		Xerostomia		Number of prescription medication categories	
		*Beta (95% CI)*	*p*	*OR (95% CI)*	*p*	*OR (95% CI)*	*p*	*OR (95% CI)*	*p*	*OR (95% CI)*	*p*	*Beta (95% CI)*	*p*
Preservation of teeth, edentulous	Unadjusted	0.05 (−1.71–1.81)	.954	0.93 (0.25–3.50)	.914	1.20 (0.41–3.49)	.741	13.43 (5.13–35.17)	<.001	1.05 (0.46–2.39)	.916	0.67 (−0.16–1.49)	.115
	Adjusted	0.29 (− 1.53–2.12)	.753	0.84 (0.21–3.36)	.809	1.08 (0.36–3.22)	.896	13.06 (4.89–34.89)	<.001	1.07 (0.45–2.55)	.877	0.79 (− 0.08–1.66)	.077
Use of professional oral health care, 0–5 years ago	Unadjusted			1.16 (0.44–3.05)	.759	1.64 (0.77–3.49)	.202			1.32 (0.70–2.47)	.391		
	Adjusted			1.36 (0.51–3.67)	.539	1.81 (0.83–3.98)	.138			1.47 (0.76–2.84)	.256		
Gender, female	Unadjusted									1.24 (0.61–2.50)	.549		
	Adjusted									1.28 (0.62–2.64)	.506		
ADL independence, BI score	Unadjusted							0.98 (0.92–1.05)	.554				
	Adjusted							0.99 (0.92–1.06)	.734				
Cognitive functioning, MMSE score	Unadjusted					0.97 (0.88–1.07)	.597						
	Adjusted					0.98 (0.88–1.08)	.623						
Xerostomia, yes	Unadjusted											1.34 (0.56–2.13)	.001
	Adjusted											1.41 (0.61–2.20)	.001

Odds ratios (OR) and 95% confidence intervals (95% CI) were derived from ordinal logistic regression and betas and 95% CI from linear regression analyses. Models were adjusted for gender, age, education

Oral pain or discomfort (no, sometimes, yes), time period since last visit to an oral health care provider (< 1 year, 1–5 years, 5–10 years and > 10 years), chewing ability (good, rather good, poor, not able) and xerostomia scores (score 5, 6, 7, 8, 9 and > 10) were entered as ordinal independent variables in the ordinal logistic regression models. Cognitive functioning (MMSE score) and number of prescription medication categories were entered as continuous independent variables in the linear regression models. ADL = Activities of daily living. MMSE = Mini-Mental State Examination

### Use of professional oral health care, preservation of teeth, and ADL independence

The majority (64%) of the dentate centenarians had visited an oral health care provider less than 1 year prior to the interview. In contrast, the majority (59%) of the edentulous centenarians had *not* visited an oral health care provider for at least 10 years prior to the interview. Indeed, 48% of the centenarians who had regularly visited an oral health care provider was dentate (14/29). While consulting an oral health care provider was associated with being dentate (*p* < .001), it was not associated with ADL independence (*p* = .734). Centenarians who visited an oral health care provider 0–5 years ago, had similar levels of oral pain or discomfort (*p* =. 539), chewing ability (*p* = .138), and xerostomia (*p* = .256) compared to centenarians who visited an oral health care provider more than 5 years ago prior to the interview.

### Xerostomia, gender, and use of prescription medications

Xerostomia scores did not differ between males and females (*p* = .506, Table 3). However, centenarians who had symptoms of xerostomia, used significantly more prescription medication compared to centenarians who had no symptoms of xerostomia (B = 1.41 [95% CI: 0.61–2.20], *p* = .001), Table 3). Medications that were most frequently used included cardiac medication, diuretics, anti-coagulants, and antacids (respectively 53, 47, 45 and 41% of the centenarians).

## Discussion

Here we described the self-reported oral health and frequency of oral health care visits in a unique cohort of 162 self-reported cognitively healthy centenarians in the Netherlands. The large majority (83%) of the centenarians were edentulous and were wearing removable complete maxillary and mandibular dental prostheses. We assume that many of the centenarians in this study have been wearing these dental prostheses for several decades. This may have led to severe atrophy of the edentulous alveolar ridges and consequently often to oral pain and discomfort. Surprisingly, 76% of the participants within the total sample did not experience any oral pain or discomfort while 49% had not visited an oral health care provider for at least 10 years.

The fraction of edentulous centenarians in the 100-plus Study cohort was higher than the edentulous centenarians from the New England Centenarian Study (NECS) [19]: 83% vs 37%. The NECS studied the oral health of 64 centenarians, of these, 35% retained more than half of the total number of natural teeth, 29% had retained less than half of the total number of natural teeth, and 67% wore removable complete or partial dental prosthesis. This difference between the two centenarian cohorts may be explained by the fact that the edentulousness in older adults in de United States is 20–30% lower compared to the Netherlands [20]. The fraction of edentulous 65–74 year-olds in the United States decreased from 45% in 1971–1974 to an estimated 24% in 1999–2004 [20]. By comparison, a 2009 survey in the Netherlands estimated that 41% of the people aged ≥65 years was edentulous [11], compared to 61% of the 80-year-olds in a community-dwelling sample in 2015 [21].

Between the ages of 60 and 70 years, the now-centenarians from the NECS had lower rates of edentulousness than their generation peers, suggesting that they had a better oral health during adulthood [19]. Reports indicated that people from the higher socio-economic classes have better oral health, both in the United States and the Netherlands [22]. Indeed, the centenarians from the NECS came from higher socio-economic classes. The centenarians from the 100-plus Study also came from higher socio-economic classes compared to their birth generation [12], and although we did not address retrospective oral health in our questionnaire, we might expect that they also had a better oral health during adulthood [12]. This may be further supported by the finding that the fraction of individuals who experienced xerostomia is considerably lower in the centenarians compared to home-dwelling 60–80-year-olds in the Netherlands (18% vs 43%, albeit that oral health questionnaires were different) [23]. Of note, the centenarians who did report symptoms of xerostomia used more prescription medication than those who did not suffer from symptoms of xerostomia [8].

Within our cohort, dentate and edentulous centenarians reported similar chewing ability, similar levels of oral pain or discomfort and xerostomia scores. The self-reported absence of oral pain or discomfort may be influenced by the optimistic nature of centenarians from the 100-plus Study [12]; centenarians across populations are more likely to be agreeable and conscientious individuals, and they may be less likely to complain [24–26]. Although we could not test this, we speculate that centenarians might avoid chewing problems or oral discomfort by preferring softer foods. In this group, we could not replicate the association between masticatory (chewing) activity and cognitive functioning as found by others [5, 7]. The inclusion criteria of the 100-plus Study include being cognitively healthy, which may obscure any relation between cognitive function.

Half of the edentulous centenarians had not visited an oral health care provider for at least 10 years prior to the interview, this is in line with the observation that 69% of the home-dwelling older adults in the Netherlands who received formal home care, had not consulted an oral health care provider since many years [27]. Older people seem to favor long-established oral hygiene routines, but with increasing frailty they fail to continue their usual oral health care behavior and stop consulting oral health care providers [28]. However, in this selected cohort of centenarians, we did not find an association between dental consultation and the level of dependency in ADL.

Although most of the centenarians within the 100-plus Study cohort were edentulous, the number of dentate centenarians will increase in the near future [11, 29]. Given our findings that dentate centenarians tend to seek more oral health care, policy makers and educators should be we aware of an increasing need for oral health care in the oldest-old. Dutch oral health care providers might at present not be capable and appropriately trained to meet these changing needs of the population. As a first step, researchers in collaboration with clinicians from the Netherlands and Belgium, now established a research agenda ‘*Oral care for elderly’*, which aims to conduct more research in this area and to expand and implement newly acquired knowledge in the near future [30].

### Limitations and future studies

We acknowledge that oral health of centenarians should ideally be objectively assessed by an oral health care provider. It might be that undetected types of oral pathology may be present beneath dental prostheses or in other parts of the oral cavity. Dental caries of periodontal disease may exist in the dentate group, but not cause pain or discomfort. Future studies including an oral health care check-up by an oral health care provider would allow the assessment of the quantity and quality of their saliva, which is important for the protection of oral health [8]. We note that this centenarian cohort represents a high-performing sub-selection of cognitively healthy Dutch centenarians, such that our findings may not be representative for the general centenarian population in the Netherlands.

## Conclusions

The large majority of the centenarians in the current study were edentulous and had not consulted an oral health care provider since many years. However, few centenarians complained of oral discomfort, pain or xerostomia, which may in part be influenced by the optimistic nature of this group. The number of dentates in the oldest-old is expected to increase in the near future. To provide suitable oral health care for the oldest-old, providers should be aware that an increasing amount of centenarians will need professional oral health care in the future.

## Data Availability

The datasets used and/or analyzed during the current study are available from the corresponding author on reasonable request.

## Abbreviations

ADL: activities of daily living
BI: Barthel Index
ISCED: International Standard Classification of Education
MMSE: Mini-Mental State Examination
NECS: New England Centenarian Study
